# Supplementary material for: Causal interplay between lactose intolerance and gut microbiota: a combined bidirectional Mendelian randomization and in vivo validation study
Source: Front Nutr. 2026 Jun 1;13:1803337. doi: 10.3389/fnut.2026.1803337 (PMC13265576; doi:10.3389/fnut.2026.1803337)
Supplement: Supplementary file 1 [file Data_Sheet_1.zip › supplementary materials/Forward/leave one out cross validation/ebi-a-GCST90027465.finngen_R12_E4_LACTONAS.pdf]

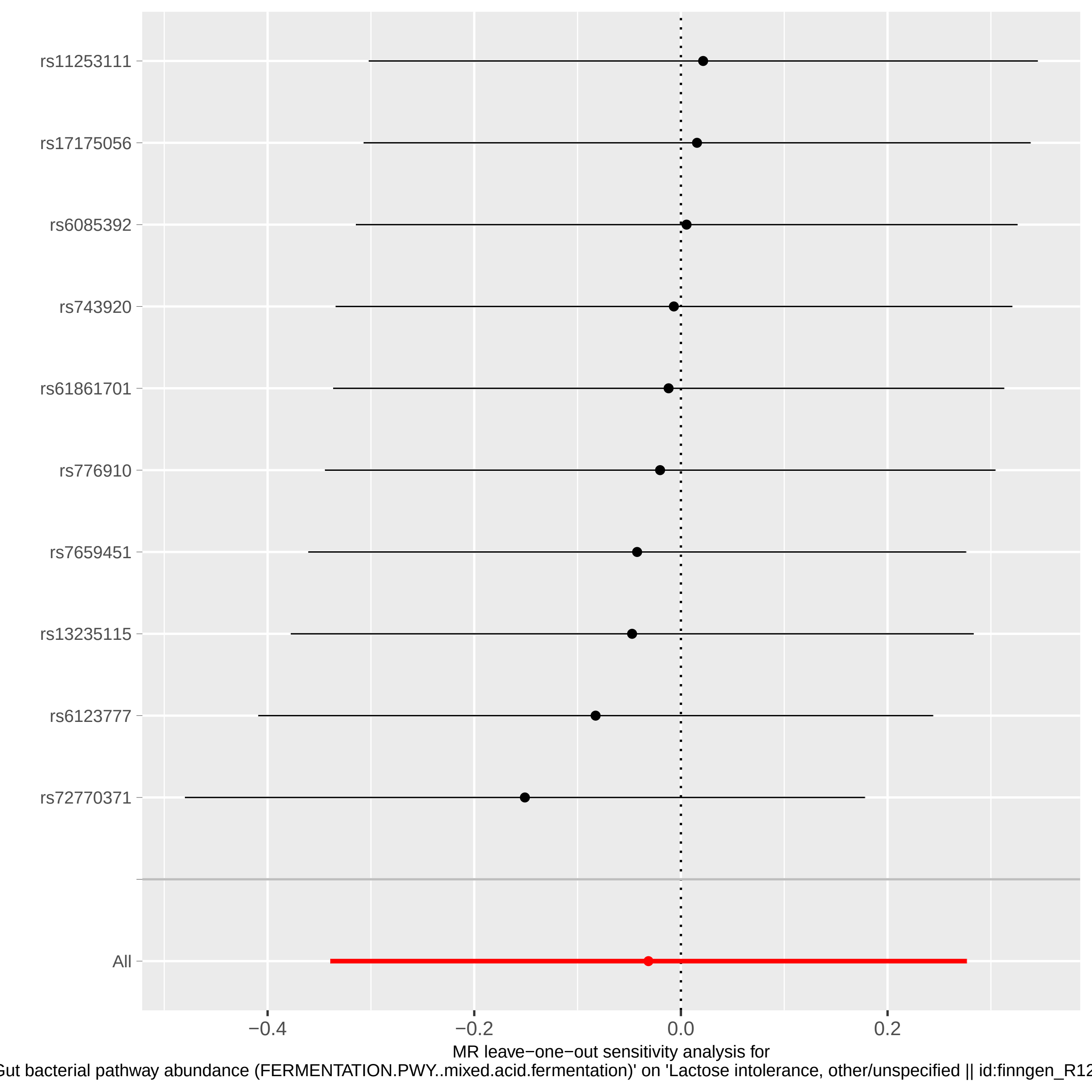

rs11253111
rs17175056
rs6085392
rs743920
rs61861701
rs776910
rs7659451
rs13235115
rs6123777
rs72770371
All
−0.4
−0.2
0.0
0.2
MR leave−one−out sensitivity analysis for
'Gut bacterial pathway abundance (FERMENTATION.PWY..mixed.acid.fermentation)' on 'Lactose intolerance, other/unspecified || id:finngen_R12
